# RARE ASSOCIATION OF HYPER IgE SYNDROME WITH CERVICAL RIB AND NATAL TEETH

**DOI:** 10.4103/0019-5154.57617

**Published:** 2009

**Authors:** Anupama S Roshan, C Janaki, B Parveen, N Gomathy

**Affiliations:** *From the Department of Dermatology, Madras Medical College, Chennai, India.*

**Keywords:** *Cervical rib*, *hyper IgE syndrome*, *natal teeth*

## Abstract

Hyper IgE syndrome (HIES) is a rare immunodeficiency syndrome characterized by a triad of cutaneous abscesses, mostly caused by *Staphylococus aureus*; pneumonia; and raised IgE levels. Nonimmunological associations include course facial features, multiple bone fractures, joint hyperextensibility, and retained primary dentition. Patients require long-term antibiotic therapy. We report here a classical case of HIES with rare associations of natal teeth, bilateral cervical ribs, and conductive deafness. The patient was being treated with monteleukast and dapsone.

## Introduction

Hyperimmunoglobulenemia E syndrome (also known as Job's syndrome or hyperimmunoglobulin E recurrent infection syndrome) is a rare syndrome presenting classically with a triad of recurrent staphylococcal skin infection, pneumonia with pneumatocele formation, and high serum levels of IgE. Other common features include an eczematous dermatitis, skeletal malformations and dental abnormalities. Most cases occur sporadically or inherited in an autosomal dominant fashion, although autosomal recessive inheritance has also been reported. We report here the case of a girl child with hyper IgE syndrome with the rare associations of natal teeth and cervical ribs.

## Case Report

A ten-year-old girl born of third-degree consanguineous parentage and uneventful pregnancy was brought with multiple itchy skin lesions and multiple swellings over the scalp. There was history of natal teeth, the upper two incisors were present at birth and multiple vesicles at various sites since three days of age. Later on, itchy skin lesions developed with multiple abscesses. Septic arthritis on the right hip joint developed at eight years of age with subsequent dislocation. There was no history of sinopulmonary infections or asthma. None of the family members were similarly affected or were asthmatic.

**Figure 1:**
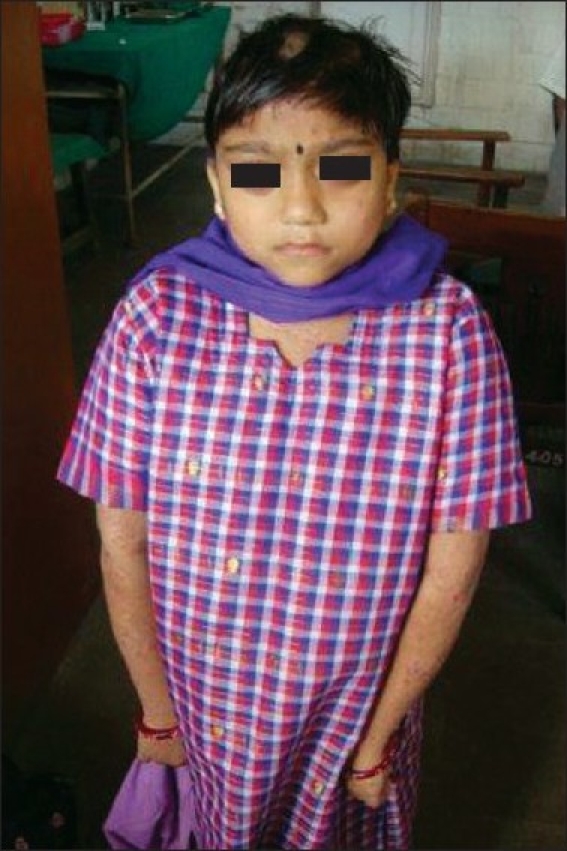
Coarse facial features with increased head circumference

**Figure 2:**
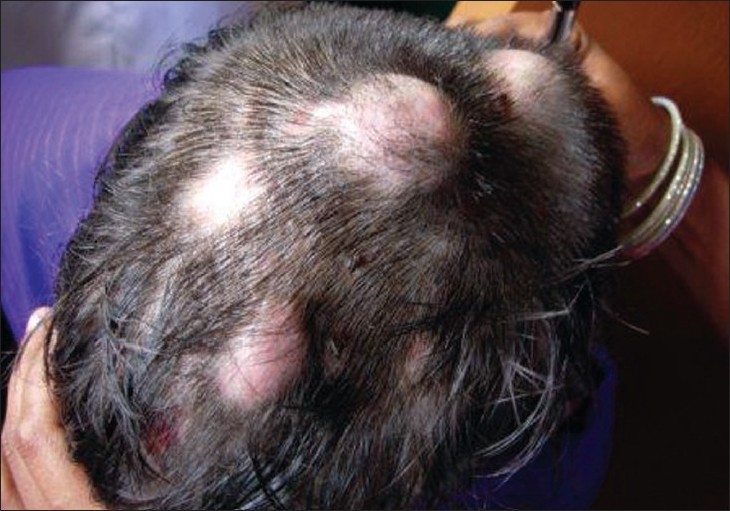
Multiple painless abscesses over the scalp

**Figure 3:**
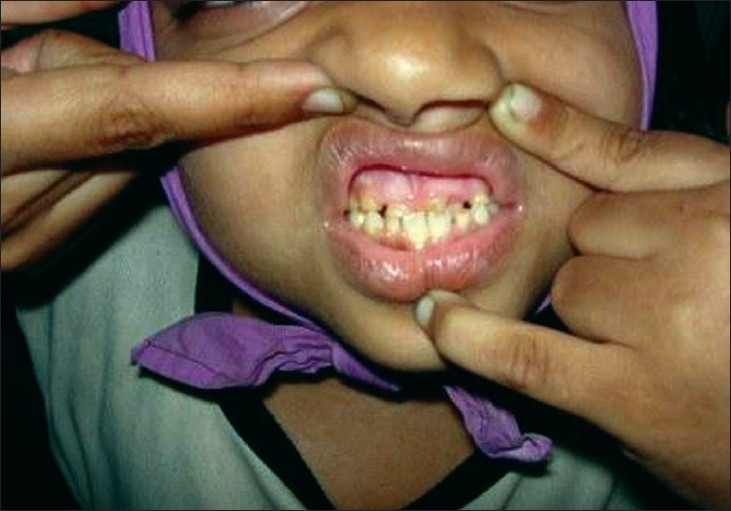
Retention of primary dentition

**Figure 4:**
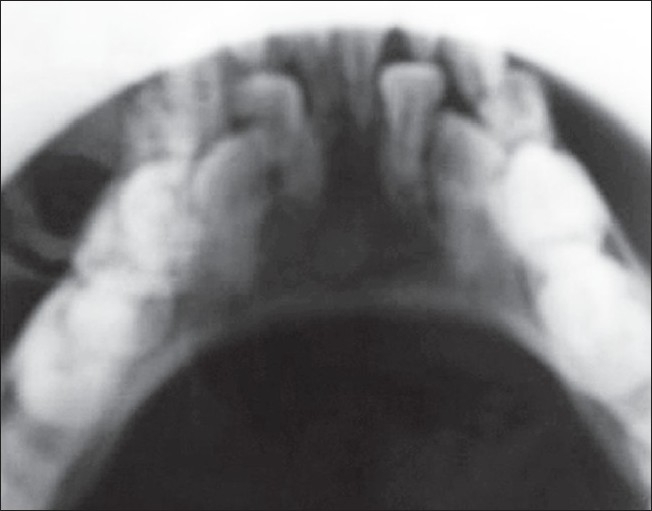
Jaw X-ray showing retention of primary dentition

On examination, the child appeared stunted and obese. She had coarse facial features with depressed nasal bridge and proptosis of right eye. Head circumference was increased [Figure 1]. She had dry skin with multiple eczematized lesions over the trunk and limbs with accentuation in auricular and retroauricular area. Multiple painless abscesses were present over the scalp [Figure 2] which on incision and drainage discharged thick creamy pus. Primary dentition of the lower two incisors was retained [Figures 3 and 4]. A scar of drainage abscess in the left axilla was present.

**Figure 5:**
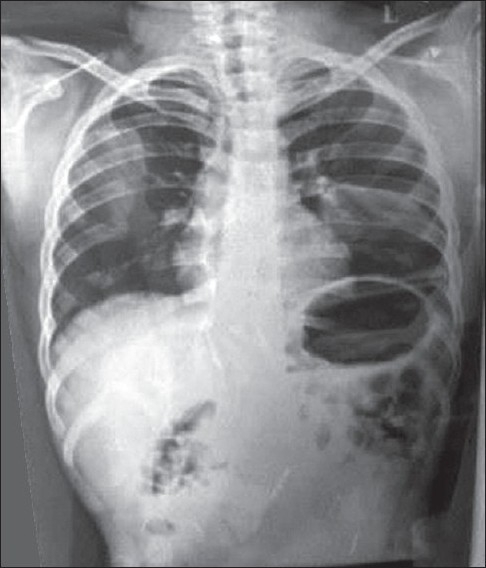
Chest X-ray revealing bilateral cervical ribs

**Figure 6:**
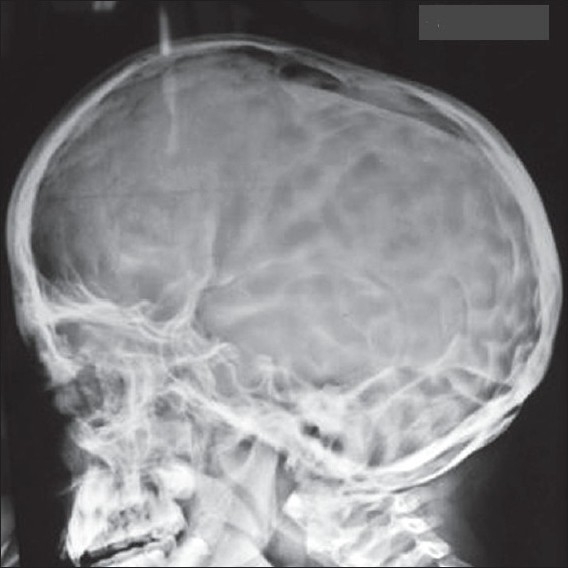
Skull X-ray showing osteopenic lesions and retained primary dentition

*Staphylococeous aureus* sensitive to amikacin and ceftrioxone was grown from the pus. Scraping of scalp revealed dermatophyte hyphae. Absolute eosinophil count was 2710 and IgE was 1,10,000. Chest X-ray revealed bilateral cervical ribs, but was otherwise clear. Skull X-ray showed osteopenic lesions and retained primary dentition [Figures 5 and 6] and hip joint X-ray showed dislocation on the right side. CT scan of the skull showed craniosynostosis with scalp abscess in the right parietal region.

Patient was started on Monteleukast, 6 mg/day, Dapsone, and oral Vitamin C. Though the incidence of infection subsided, itching did not regress.

## Discussion

HIES is a multisystem disorder characterized by recurrent staphylococcal lung abscesses, pneumonia with pneumatocoele formation, and elevated IgE levels of > 2000 IU.[1] The pathophysiology of Job's syndrome is not completely understood. Earlier reports described a chemotactic defect in neutrophils. This has been attributed to defective production of IFN-γ, which is a major stimulator of neutrophils, when stimulated by IL-2. The increase in IgE levels occur by means of unopposed IL-4 action.

The cutaneous abscesses occur more commonly on the head and neck and in the intertriginous areas. Vesicular eruptions similar to herpetic lesions may also be present in the neonatal period which later become eczematized and lichenified.[2] Bacterial septic arthritis is also commonly present. Other nonimmunological features, include retention of primary dentition, and scolosis. Characteristic facies shows greater inter alar width and longer outer canthal distance. A prominent brow and supraorbital ridge with impression of deep-set eyes is also observed.[2] Other features that can present include otitis externa, otitis media, and sinopulmonary infections.

Dental anomalies include retained primary dentition, noneruption of permanent teeth, and double rows of teeth with both primary and permanent intermixed teeth.[2] Delayed dentition was reported in 75% in a study conducted by OConnell *et al.*[3] Skeletal anomalies include scoliosis, wedge-shaped L2 vertebra, hemivertebra, spina bifida, bifid rib, and pseudoarthrosis of rib. Hyperextensible joints including fingers, wrist, shoulders, hips, and knees may also be present.[1]

Our patient typically had a course facies, cold abscesses over the scalp and axilla, septic arthritis, IgE levels of 1,10,000, and absolute eosinophil count of 2710. In addition, she also had bilateral cervical ribs. Cervical ribs are present in 0.5–0.1% of the total population and are twice as common in females as in males.[4] Since the incidence is rare in the general population cervical rib can be considered a rare association of Job's syndrome. She also had natal teeth. In the general population the incidence is very rare varying from one in thousand to one in thirty thousand.[5] It is commonly present in syndromes like Ellis-van Creveld syndrome, and Hallerman-Strieff syndrome. Since other features of these syndromes are not present in our case, this too could be considered a rare association of Job's syndrome. Conductive deafness may probably be attributed to otitis media which went unnoticed. She did not have any other systemic involvement.

Treatment of HIES involves giving appropriate antibiotics and drainage of abscesses. Other treatment options include levamisole, cimetidine, ascorbic acid, and transfer factor. Methotrexate can also be tried.[6]
